# Removal of trace metal contaminants from potable water by electrocoagulation

**DOI:** 10.1038/srep28478

**Published:** 2016-06-21

**Authors:** Joe Heffron, Matt Marhefke, Brooke K. Mayer

**Affiliations:** 1 Marquette University, Department of Civil, Construction and Environmental Engineering, Milwaukee, WI 53233, USA

## Abstract

This study investigated the effects of four operational and environmental variables on the removal of trace metal contaminants from drinking water by electrocoagulation (EC). Removal efficiencies for five metals (arsenic, cadmium, chromium, lead and nickel) were compared under varying combinations of electrode material, post-treatment, water composition and pH. Iron electrodes out-performed aluminum electrodes in removing chromium and arsenic. At pH 6.5, aluminum electrodes were slightly more effective at removing nickel and cadmium, while at pH 8.5, iron electrodes were more effective for these metals. Regardless of electrode, cadmium and nickel removal efficiencies were higher at pH 8.5 than at pH 6.5. Post-EC treatment using membrane filtration (0.45 μm) enhanced contaminant removal for all metals but nickel. With the exception of lead, all metals exhibited poorer removal efficiencies as the ionic strength of the background electrolyte increased, particularly in the very high-solids synthetic groundwaters. Residual aluminum concentrations were lowest at pH 6.5, while iron residuals were lowest in low ionic strength waters. Both aluminum and iron residuals required post-treatment filtration to meet drinking water standards. EC with post-treatment filtration appears to effectively remove trace metal contaminants to potable water standards, but both reactor and source water parameters critically impact removal efficiency.

Geologic and anthropogenic heavy metals contaminate drinking water for millions of people worldwide^1^,^2^. Electrocoagulation (EC)–the *in situ* generation of coagulant by electrolytic oxidation of metal electrodes–is a century-old process gaining new traction for metal removal from water and wastewater. EC can achieve greater than 70% removal of dissolved organic carbon^3^,^4^ and 99% turbidity removal^5^. One benefit of EC is the ability to remove a wide variety of contaminants by a single process^6^. Unlike other sorption technologies like ion exchange, EC can remove both cationic and anionic metal species^7^. In addition, recalcitrant metal species such as arsenite and dichromate can be removed without prior chemical treatment^8^,^9^,^10^. However, the low conductivity of drinking water and low target contaminant concentrations required for human consumption present challenges for using EC to treat drinking water. For this reason, much of the research on metal contaminant removal by EC has used contaminant concentrations and water matrices applicable to wastewater rather than potable water^3^.

Previous EC experiments have demonstrated promise for removal of metal contaminants to below regulatory levels for drinking water^10^,^11^,^12^,^13^. Studies have also indicated that many individual factors contribute to effective metal removal by EC. For the recalcitrant ions arsenite and dichromate, iron electrodes in particular have been found to facilitate oxidation to arsenate and reduction to chromic ions, respectively^8^,^9^,^10^,^14^,^15^. Other studies have shown aluminum electrodes to be effective in removing metal cations such as nickel, copper and zinc^11^,^12^,^16^,^17^. Post-treatment filtration has also been shown to provide additional contaminant removal compared to gravitational separation^15^.

However, the wide range of operating parameters used in existing reports makes comparison between studies difficult. In addition, electrolytes in the water matrix, such as calcium, magnesium, bicarbonate and chloride ions, have been shown to influence floc formation^16^,^18^,^19^. Previous reports have used either a single natural water source^9^,^11^,^12^,^13^ or simple electrolyte solutions^16^,^20^,^21^. Thus, the wide variation in natural waters has not been examined. Moreover, the literature lacks a systematic examination of multiple EC parameters for the removal of a suite of metal contaminants. Discerning interactions between variables, *e.g.,* the comparative benefits of different electrode materials at different pH levels, is particularly problematic. By simultaneously varying operational and water quality parameters, this study examines the relative importance of, and interactions between, these critical variables.

In addition to removing contaminant metals, EC treatment units should also meet drinking water standards for residual concentrations of the coagulant metal. While some researchers have alluded to challenges with coagulant residuals^22^,^23^, a systematic investigation is lacking as to how operating and environmental conditions influence these residuals. Aluminum and iron have secondary Maximum Contaminant Levels (MCLs) for drinking water of 0.2 and 0.3 mg/L, respectively^24^. Though these U.S. Environmental Protection Agency standards are non-enforceable, they are nonetheless important for successful implementation of water treatment technology. Iron levels above 0.3 mg/L discolor drinking water and stain laundry and plumbing^25^. In addition to aesthetic concerns, chronic ingestion of aluminum in drinking water may have neurotoxic effects^26^.

The goal of this research was to determine the effects and relative importance of reactor and water quality parameters on metal removal from a mixed-contaminant stream using EC. Five metals of concern for drinking water contamination (arsenic, cadmium, chromium, nickel and lead) were simultaneously spiked at low levels typical of contaminated drinking water, as shown in Table 1. Four critical EC parameters were varied: electrode material, post-treatment, pH and water matrix composition. Both aluminum and iron electrodes were tested. Treated waters were subjected to two forms of post-treatment: 0.45 μm membrane filtration or settling alone. In addition, the initial pH value of the test water was adjusted to either 6.5 or 8.5. Rather than varying a single background ion or using a single natural water, four synthetic waters were prepared to represent the ionic composition of a wide range of natural waters. Contaminant metal removal efficiencies, as well as residual concentrations of aluminum and iron, were compared across the range of parameters.

## Results and Discussion

### Electrode material

**Figure 1:**
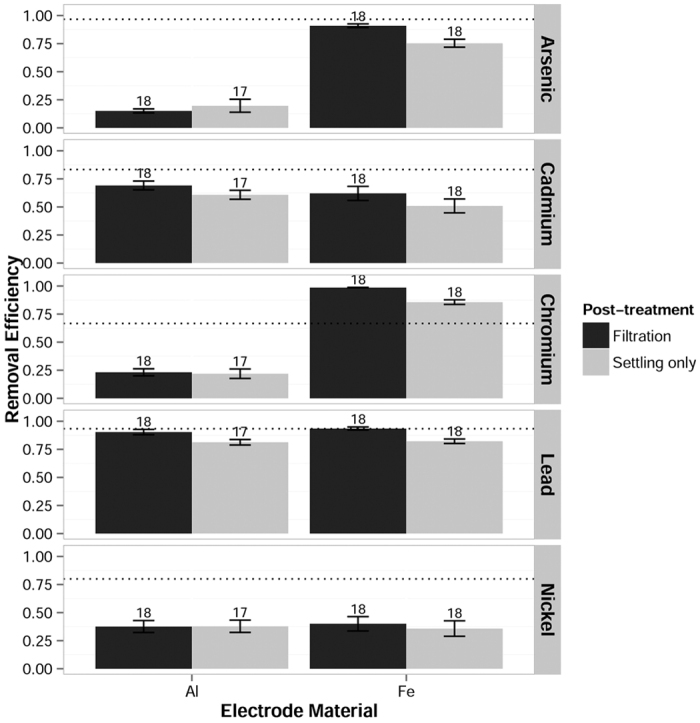
Removal efficiencies of five metals grouped by post-treatment and electrode material. Iron electrodes were vastly superior to aluminum for removing arsenic and chromium (spiked as arsenite and dichromate, respectively). Post-treatment filtration (0.45 μm) significantly increased cadmium and lead removal efficiencies over settling alone. Filtration also enhanced arsenic and chromium removal, but only with iron electrodes. The dotted line represents the removal efficiency necessary to meet the MCL for each metal (MDWL for nickel). Error bars represent the standard error of the group mean. Values for n are shown above each bar.

Electrode material was the most significant factor determining arsenic and chromium removal. Iron electrodes drastically outperformed aluminum electrodes for both arsenic (F(1, 66) = 265, p ≈ 0) and chromium (F(1, 64) = 295, p ≈ 0), as shown in Fig. 1. Since both contaminants were spiked in their more recalcitrant forms (arsenite and dichromate), the greater removal observed with iron electrodes may be due to redox conversion of the contaminants. Previous research^8^,^10^ found that reactive iron species generated by electrocoagulation can convert As(III) to As(V) and Cr(VI) to Cr(III). The oxidized arsenic and reduced chromium species are more easily removed from solution by sorption or precipitation than are arsenite and dichromate^8^,^9^,^10^.

**Figure 2:**
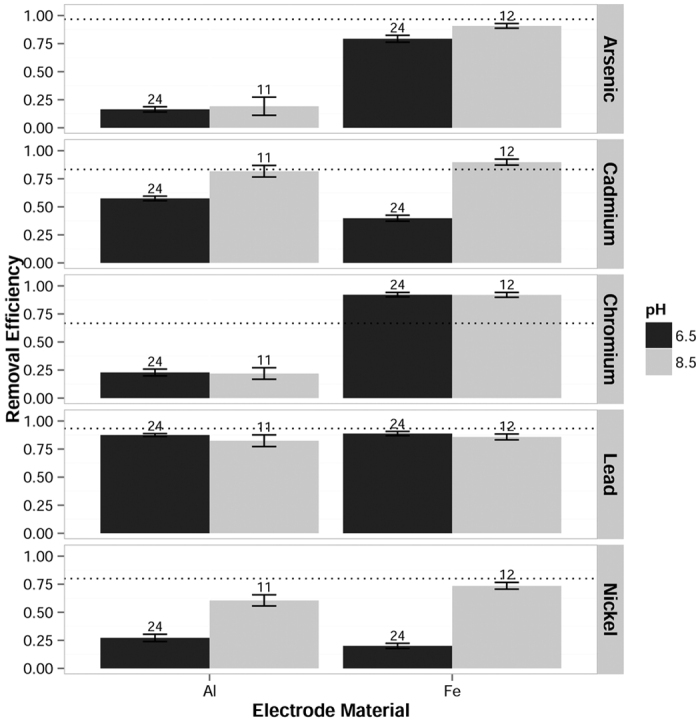
Removal efficiencies of five metals grouped by pH and electrode material. Only cadmium and nickel removal was significantly affected by pH, with greater removal at pH 8.5 than pH 6.5. At pH 6.5, greater removal of cadmium and nickel was observed with aluminum iron electrodes; at pH 8.5, greater removal was seen with iron electrodes. The dotted line represents the removal efficiency necessary to meet the MCL for each metal (MDWL for nickel). Error bars represent the standard error of the group mean. Values for n are shown above each bar.

For cadmium and nickel, iron electrodes performed slightly better than aluminum at pH 8.5, and slightly poorer than aluminum at pH 6.5, as shown in Fig. 2. This interaction between electrode material and pH was significant for both cadmium (F(1, 64) = 12.3, p = 0.000824) and nickel (F(1, 66) = 10.7, p = 0.00171). The advantage of aluminum at pH 6.5 and iron at pH 8.5 may stem from the comparative insolubility of Al^3+^ and Fe^3+^ ions at those pH levels. In addition, aluminum electrodes may provide better cation removal at pH 6.5 because soluble aluminum hydroxide species are predominately anionic near neutral pH, while soluble iron (III) hydroxides have a predominantly positive charge^27^. Additional research would be required to verify this hypothesis.

### Post-treatment

Post-treatment filtration enhanced removal of arsenic, cadmium, chromium and lead over settling alone. The effect of post-treatment was significant for both cadmium (F(1, 64) = 15.9, p = 0.000173) and lead (F(1, 68) = 25.6, p = 3.41 × 10^−6^). For arsenic and chromium, filtration only enhanced removal when using iron electrodes, as shown in Fig. 1. The interaction between electrode material and post-treatment was significant for both arsenic (F(1, 66) = 9.43, p = 0.00310) and chromium (F(1, 64) = 6.28, p = 0.0147). The concentration of arsenic and chromium on aluminum-entrained flocs may have been too low to register additional polishing by filtration. Similarly, nickel removal was poor compared to other contaminants and did not show a significant effect from filtration. According to visual observations, iron formed small flocs that readily re-suspended while decanting, while aluminum coagulants formed larger, more cohesive flocs. Thus, the need for additional filtration to remove iron flocs was anticipated.

### Water matrix

Contaminant removal was tested in four different synthetic waters representing surface or groundwater with low or high conductivity (SL = surface low, SH = surface high, GL = ground low, GH = ground high). Contaminant metals were spiked at challenge concentrations listed in NSF/ANSI 53-2011a^28^, as shown in Table 1. The NSF/ANSI protocol did not provide a challenge concentration for nickel, so a spiking concentration of five times the Maximum Drinking Water Level was chosen.

**Figure 3:**
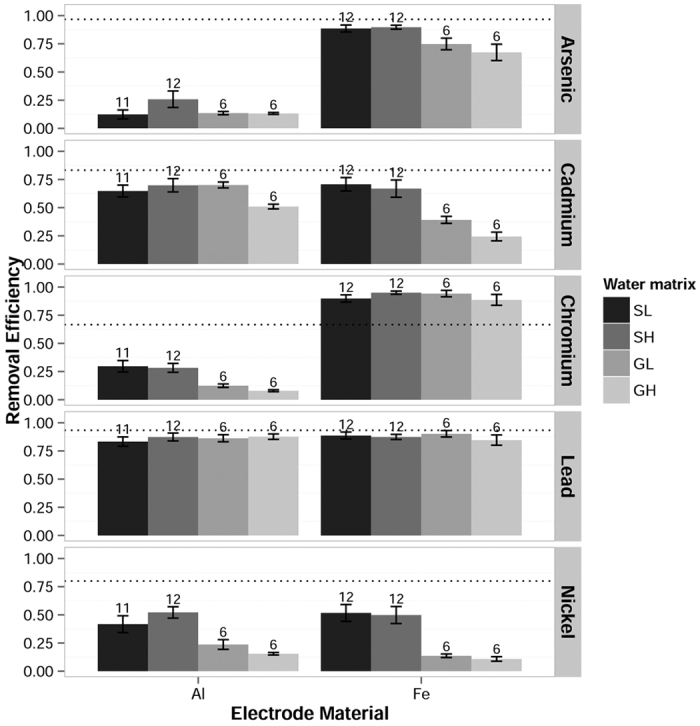
Removal efficiencies of five metals grouped by water matrix and electrode material. Water matrices varied in composition to mimic low and high ionic strength surface and ground waters: surface low (SL), surface high (SH), ground low (GL) and ground high (GH). Arsenic, cadmium, chromium and nickel removal decreased with increasing ionic strength. For chromium, this trend was less evident with iron electrodes, though still significant. For cadmium, the trend was only significant with iron electrodes. Lead removal was consistently high in all waters. The dotted line represents the removal efficiency necessary to meet the MCL for each metal (MDWL for nickel). Error bars represent the standard error of the group mean. Values for n are shown above each bar.

For equivalent charge loading, most metals showed decreasing removal efficiency with increasing conductivity of the water matrix, as shown in Fig. 3. The main effect of conductivity was significant for arsenic (F(1, 66) = 9.58, p = 0.00289), chromium (F(1, 64) = 28.2, p = 1.49 × 10^−6^) and nickel (F(1, 66) = 16.8, p = 0.000114). Chromium removal was less inhibited by increased conductivity when iron electrodes were used, with a significant interaction between electrode material and conductivity (F(1, 64) = 8.00, p =0.00623). Conversely, cadmium removal was only significantly inhibited by increased conductivity while using iron electrodes (F(1, 64) = 9.89, p = 0.00252).

Chromium removal efficiencies with iron electrodes were high (77 to 99%) in all test conditions. Other effects, such as those of ionic strength, were likely dampened as measurements approached the method detection limit (11.0 μg-Cr/L). Similarly, lead was consistently removed to concentrations below the MCL, and lead removal showed no significant effect from changes in conductivity. These findings indicate that electrocoagulation efficiently mitigates chromium and lead under a wide variety of conditions.

The four water matrices were intended to mimic composition changes in natural waters that accompany increased ionic strength. Therefore, the effects associated with change in conductivity should not be attributed to ionic strength alone. However, this experimental method provides a more accurate model of EC treatment of natural waters than would a comparison of different concentrations of a uniform solution. Other constituents in the water matrix may impact metal removal as well. Complexation with ligands other than those considered here, *e.g.*, NH_3_, may also impact metal solubility and charge^29^. In addition, both coagulant ions and trace metals may bind to natural organic matter in the water matrix^30^. Future studies assessing the particular effects of these variable constituents can be compared to the baseline of EC performance determined in this study, which uses characteristic, universal water constituents.

**Figure 4:**
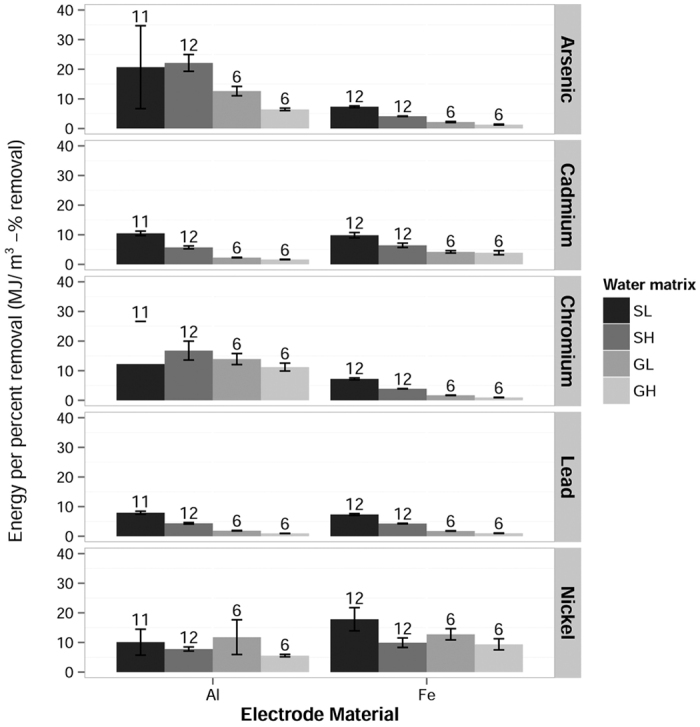
Energy required per percent contaminant removal per cubic meter of water treated. Despite generally poorer removal efficiency for equivalent charge loading (as shown in Fig. 3), higher ionic strength waters required less energy to reduce contaminant concentrations by one percent. Error bars represent the standard error of the group mean. Values for n are shown above each bar.

The effect of conductivity was most apparent in very high ionic strength water matrices. This is relevant because many geologically contaminated groundwaters have very high concentrations of dissolved solids^31^. Though contaminant removal was lower for the given charge loading, passing charge through high conductivity waters requires less power. Based on the treatment levels in this study, high ionic strength waters required less energy per percent contaminant removal than low solids waters, as shown in Fig. 4. High conductivity waters may therefore prove superior candidates for EC based on equivalent power consumption. Further research is required to determine if the maximum contaminant removal in high ionic strength waters can match that of low ionic strength waters, and whether high ionic strength waters continue to be more energy efficient when removing contaminants to below drinking water standards. While optimizing the EC process for power consumption was beyond the scope of this work, previous researchers have suggested that the energy demands of EC could be met with photovoltaic cells^32^,^33^ or offset by harvesting hydrogen gas from the EC process^34^.

### pH

For cadmium and nickel removal, pH was the most significant factor, as shown in Fig. 2. Removal at pH 8.5 was greater than at pH 6.5 for both cadmium (F(1, 64) = 33.9, p = 2.05 × 10^−7^) and nickel (F(1, 66) = 33.5, p = 2.18 × 10^−7^). Cadmium and nickel removal at pH 6.5 was also slightly poorer with iron compared to aluminum electrodes, but removal at pH 8.5 was greater with iron electrodes. This interaction between electrode material and pH was significant for both cadmium (F(1, 64) = 12.3, p = 0.000824) and nickel (F(1, 66) = 10.7, p = 0.00171). Although chromium and lead may have had slightly better removal at pH 6.5 than pH 8.5, the main effect of pH did not quite achieve 95% confidence for either chromium (F(1, 64) = 3.96, p = 0.0508) or lead (F(1, 68) = 3.75, p = 0.0570). Both chromium and lead were consistently removed to very low concentrations in a subset of the tests. The effect of pH may have been difficult to distinguish at this level of removal, as previously mentioned in the discussion of water matrix effects.

Initial cadmium and nickel concentrations in filtered samples of the test waters show that both metals remained soluble at spiked concentrations throughout this pH range. Since both aluminum and iron electrodes were shown to be more effective at pH 8.5 for cadmium and nickel, decreased coagulant solubility is an unlikely cause for greater removal. Cadmium and nickel cations may show greater affinity to the more negatively charged aluminum and iron species at high pH. In addition, previous experiments have shown that calcium and magnesium cations precipitate as carbonate species at the cathode surface^35^,^36^. Cadmium and nickel cations may share a similar fate, although this hypothesis requires additional research.

### Residual aluminum and iron

**Figure 5:**
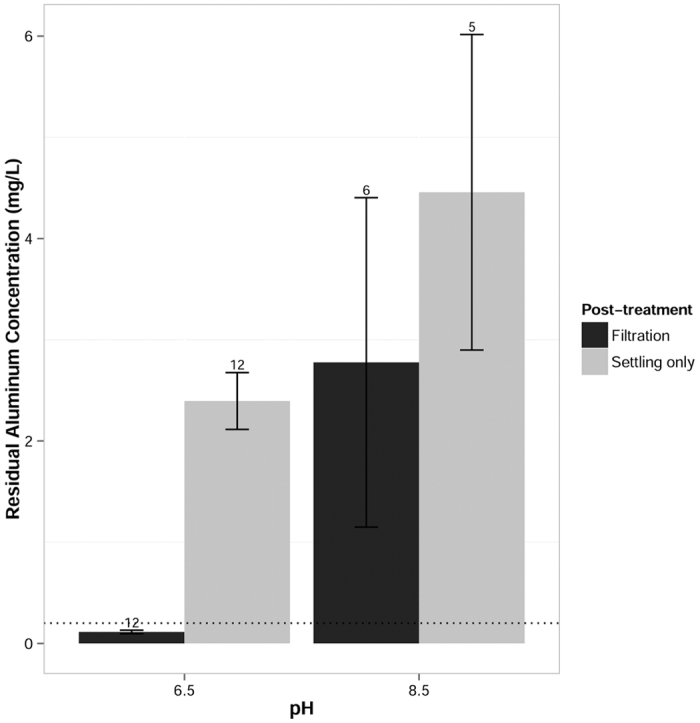
Concentrations of residual aluminum in treated water. Residual aluminum concentrations were significantly lower at pH 6.5 than pH 8.5. Post-treatment filtration (0.45 μm) significantly reduced aluminum residuals over settling alone. The dotted line represents the secondary MCL for aluminum. Error bars represent the standard error of the group mean. Values for n are shown above each bar.

**Figure 6:**
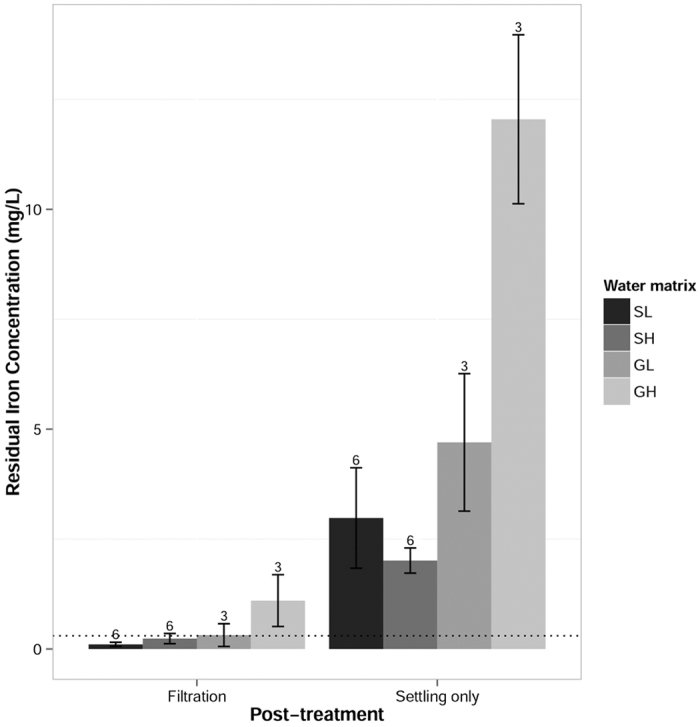
Concentrations of residual iron in treated water. Water matrices varied in composition to mimic low and high ionic strength surface and ground waters: surface low (SL), surface high (SH), ground low (GL) and ground high (GH). Iron residuals increased with the ionic strength of the source water. Post-treatment filtration (0.45 μm) significantly reduced iron residuals over settling alone. The dotted line represents the secondary MCL for iron. Error bars represent the standard error of the group mean. Values for n are shown above each bar.

The residual concentration of aluminum or iron from the EC process varied with respect to post-treatment and water quality. Aluminum and iron have non-enforceable, secondary MCLs of 0.2 and 0.3 mg/L, respectively (US EPA, 2009). Filtration was necessary to reduce residual aluminum or iron to below these secondary MCLs. However, filtration only reduced residual aluminum to below the secondary MCL at pH 6.5, as shown in Fig. 5. The residual iron concentration increased with increasing ionic strength of the water matrix, as shown in Fig. 6. The effect of post-treatment filtration was significant for both aluminum (F(1, 32) = 8.80, p = 0.00566) and iron (F(1, 33) = 28.2, p = 7.39 × 10^−6^). However, the effect of pH was only significant for aluminum (F(1, 32) = 9.82, p = 0.00368), and the effect of conductivity was only significant for iron (F(1, 33) = 23.8, p = 2.64 × 10^−5^). Aluminum solubility changes more drastically between pH 6.5 and 8.5 than does iron (III) solubility^27^. Previous research^19^ found that iron forms larger, more ordered flocs in the presence of calcium and magnesium. These ordered solids should have a lower solubility than less crystalline solids^37^. Therefore, the higher concentration of soluble iron is likely due to iron speciation with background ions in the high ionic strength water.

At pH 8.5, aluminum electrodes are not able to meet secondary standards for residual metal concentrations. Iron electrodes do not function ideally in very high conductivity waters but may still be the preferred alternative in light of aluminum’s potential neurotoxicity^26^. However, if excessive iron reduced the aesthetic quality of treated water, users might be reluctant to adopt the technology.

## Conclusions

This research approached EC from a process perspective to demonstrate the relative importance of electrode material, post-treatment, and source water characteristics for treatment of trace metal contamination in drinking water. The impact of these parameters on removal efficacy varied among contaminants. For arsenic and chromium removal, electrode material was the most important factor, while pH was the most important factor for cadmium and nickel removal. Lead was insensitive to most experimental factors, which was likely due to consistently high removal efficiencies. However, post-treatment filtration did result in significantly lower lead concentrations.

This research suggests that electrode material should be selected first based on the contaminant; iron electrodes far exceed aluminum electrodes in removal of arsenite and dichromate, regardless of other conditions. Additionally, in high pH drinking water, iron should be sought as an alternative to aluminum electrodes for reasons of both effectiveness and meeting secondary standards for residual coagulant concentrations. Post-treatment using 0.45 μm filtration is recommended as a polishing step for reducing contaminant concentrations. Filtration was also necessary for achieving secondary MCLs for aluminum and iron.

When EC is used to treat high ionic strength waters, less removal for equivalent charge loading is achieved. Conversely, in low ionic strength waters, poor power efficiency is tempered by a higher, per-coulomb removal efficiency. Additional research is needed to investigate the tradeoff between contaminant removal efficiency and power consumption.

## Methods

### Reactor design and operation

EC tests were conducted in a 300 mL plastic batch reactor. The reactor was stirred at 60 rpm with a magnetic stir bar. Plate electrodes consisting of aluminum (6061 alloy) or iron (1120 steel) were used for both the anode and cathode. The submerged surface area of each electrode face was 54 cm^2^. The inter-electrode distance was 1 cm. Power was supplied by a 330 W direct current power supply (Sorensen LH 110-3, San Diego, CA) with a constant current density of 9.2 mA/cm^2^ (100 C/L-min). The retention time for all tests was 2 min, resulting in a charge loading of 200 C/L. This charge loading corresponded to a bulk solution concentration of 27 mg/L Al or 21 mg/L Fe. Electrode polarity was alternated every 30 seconds to prevent electrode passivation. The reactor apparatus, electrodes and polarity-alternating controller were kindly provided by A.O. Smith Corporation. Aluminum and iron electrodes were each tested in triplicate for all test water formulations.

### Test water formulation

Four synthetic test waters were prepared by spiking ultrapure (Milli-Q) water with American Chemical Society (ACS) grade reagents to approximate major ion concentrations in representative source waters, as detailed in the Supplemental Material. The test waters modeled low and high ionic concentrations for both surface and groundwater. The progression from lowest to highest ionic strength was as follows: surface low (SL, μ = 0.003 M), surface high (SH, μ = 0.006 M), ground low (GL, μ = 0.018 M), and ground high (GH, μ = 0.039 M). For each test water, calcium, magnesium, bicarbonate and sulfate concentrations were matched as closely as possible to the source water modeled (as described in the Supplemental Material). To verify that the contaminants would not co-precipitate or otherwise interact, chemical equilibrium modeling of contaminant metals in each of the four water matrices was performed with MINEQL+, version 4.6 (data not shown). Conductivity was measured prior to EC treatment using a digital Pure H_2_O conductivity meter (VWR, Radnor, PA).

The initial pH was adjusted to either 6.5 or 8.5 by addition of HCl or NaOH, respectively. Initial and final measurements of pH were recorded with an Orion 4-star pH meter (Thermo Scientific, Waltham, MA). The pH of the solution did not change over the 2 minute treatment time. Contaminant removal in SL and SH waters was tested at both pH 6.5 and pH 8.5. GL and GH waters were tested only at pH 6.5 because equilibrium models showed that lead solubility was more variable in highly alkaline waters near neutral pH (data not shown). Equilibrium models of the other metals did not show significant differences in solubility between the four waters in the pH range tested.

### Post-treatment and analysis

After EC treatment, the reactor was stirred gently to homogenize the floc suspension and 150 mL of the treated solution was transferred to 50 mL centrifuge tubes. To minimize process time, settling was simulated by centrifugation for 5 minutes at 2,910 × g, similar to the process used by Matsui, *et al.*^38^. The supernatant was then decanted. Approximately half of the supernatant was filtered through a 0.45 μm cellulose nitrate filter (Whatman, Maidstone, UK). The initial, untreated water was also filtered to determine the soluble contaminant concentrations.

Samples were digested in HNO_3_ according to Standard Method 3030 E^39^, then diluted in 2% HNO_3_ (EMD Millipore, Billerica, MA) and 0.5% HCl (BDH, Poole Dorset, UK) for trace metals analysis. Contaminant and coagulant metal concentrations were analyzed by inductively coupled plasma mass spectrometry (ICP-MS, Agilent 7700 series, Santa Clara, CA) according to Standard Method 3120 B^39^. Samples were calibrated and analyzed with Mass Hunter Workstation software, version B.01.01.

Contaminant concentrations in the treated samples were compared to filtered samples of untreated, spiked water. Models for removal of each contaminant metal were created by Generalized Least Sum (GLS) regression. A summary of the models may be found in the Supplemental Material. Analysis of Variance (ANOVA) using marginal sums of squares was used to analyze the fitted models. Computations were performed in the R language^40^ and using the ‘nlme’ package for GLS and ANOVA analyses^41^.

## Additional Information

**How to cite this article**: Heffron, J. *et al.* Removal of trace metal contaminants from potable water by electrocoagulation. *Sci. Rep.* **6**, 28478; doi: 10.1038/srep28478 (2016).

## Supplementary Material

Supplementary Information

## Figures and Tables

**Table 1:** Contaminant spiking concentrations and regulatory limits.

Species	Spiking Compound	Influent Concentration (μg/L as element)	Maximum Contaminant Level* (MCL) (μg/L)
As	AsNaO_2_	300	10
Cd	CdCl_2_	30	5
Cr	KCr_2_O_7_	300	100
Ni	NiCl_2_∙6H_2_O	500	100**
Pb	Pb(NO_3_)_2_	150	10

*As defined by the US Environmental Protection Agency^24^.

**Maximum Drinking Water Level (MDWL), not MCL^28^.
