# Supplementary material for: Removal of trace metal contaminants from potable water by electrocoagulation
Source: Sci Rep. 2016 Jun 21;6:28478. doi: 10.1038/srep28478 (PMC4914840; doi:10.1038/srep28478)
Supplement: Supplementary Information [file srep28478-s1.doc]

**Supplemental Information**

**for**

**Removal of trace metal contaminants from potable water by electrocoagulation**

**Joe Heffron^1^, Matt Marhefke^1^, Brooke K. Mayer^1*^**

^1^ Marquette University, Department of Civil, Construction and Environmental Engineering,

Milwaukee, WI 53233, USA

*Corresponding Author: Brooke.Mayer@marquette.edu

**A. Synthetic water matrices**

The reagents and quantities used to formulate each of the four test waters used in this study are provided in Table A.1. "Surface low" and "surface high" test waters were formulated to represent a range of surface water conditions based on parameters for metal contaminant removal conditions required by NSF/ANSI 53:2011a^1^. However, to allow comparison between metals, two composite surface waters were created to approximate many of the different "challenge" waters for individual metals outlined in the NSF/ANSI protocol. In addition, the low solids water outlined by the protocol was not a realistic candidate for treatment by electrocoagulation (EC) due to the very low total dissolved solids (TDS) concentration (< 100 mg/L TDS) stipulated. The extremely low conductivity of the water specified in the protocol required over 100 V of applied potential to pass 0.5 A of current, and initial tests showed that the water heated rapidly in the reactor. Thus, "surface low" was modeled to require less than 50 V, while aligning as closely as possible to the characteristics outlined in NSF/ANSI 53:2011a. "Ground low" test water was modeled on well water in Dayton, Ohio, as characterized in Snoeyink and Jenkins^2^. "Ground high" test water was modeled on brackish water from a well in Delhi, India. Sampling data for the well was kindly provided by A.O. Smith Corporation.

**Table A.1**: Composition of the four water matrices used in this study. Ion concentrations are in mmol-L^-1^.

| Synthetic Water Matrix | Mg ^2+^ | Cl - | Ca ^2+^ | SO_4_ ^2-^ | Na ^+^ | HCO_3_ - |
| --- | --- | --- | --- | --- | --- | --- |
| Surface Low (SL) | 0.181 | 1.03 | 0.399 | 0.067 | 1.20 | 1.20 |
| Surface High (SH) | 0.333 | 2.00 | 0.898 | 0.229 | 1.95 | 1.95 |
| Ground Low (GL) | 1.40 | 5.64 | 2.30 | 0.874 | 5.56 | 5.56 |
| Ground High (GH) | 1.78 | 18.9 | 2.92 | 2.10 | 22.8 | 9.09 |

1. **Regression models**

Tables B.1 and B.2 summarize the Generalized Least Sum (GLS) regression models used to evaluate the effects of the variables studied. F- and p- values were determined by ANOVA with marginal sum of squares. Conductivity (G) is a continuous value in mS/cm. All other variables are categorical variables. The default value for each categorical variable is indicated in the key below each table. For clarity, only the significant results are shown.

**Table B.1**: Regression models for metal removal efficiency as a fraction of initial, soluble metal concentration. Plus and minus values for effect sizes represent 95% confidence intervals. Shaded regions represent effects that were very nearly significant (0.05 < α < 0.06).

| Metal |  | Intercept | E_Fe_ | P_settling_ | H_6.5_ | G (mS/cm) | E_Fe_P_settling_ | E_Fe_H_6.5_ | E_Fe_ G |
| --- | --- | --- | --- | --- | --- | --- | --- | --- | --- |
| As | Effect size | 0.20 ±0.073 | 0.76 ±0.092 |  |  | -0.05 ±0.031 | -0.20 ±0.13 |  |  |
|  | F(1, 66) | 30.1 | 265 |  |  | 9.58 | 9.43 |  |  |
|  | p | 7.11x10^-7^ | 0 |  |  | 0.00289 | 0.0031 |  |  |
| Cr | Effect size | 0.28 ±0.061 | 0.69 ±0.078 |  | 0.055 ±0.055 | -0.090 ±0.033 | -0.12 ±0.092 |  | 0.066 ±0.045 |
|  | F(1, 64) | 22.8 | 295 |  | 3.96 | 28.2 | 6.28 |  | 8.00 |
|  | p | 1.07x10^-5^ | 0 |  | 0.0508 | 1.49x10^-6^ | 0.0147 |  | 0.00623 |
| Cd | Effect size | 0.86 ±0.063 | 0.11 ±0.082 | -0.092 ±0.045 | -0.23 ±0.076 |  |  | -0.19 ±0.11 | -0.080 ±0.049 |
|  | F(1, 64) | 0.49 | 11.3 | 15.9 | 33.9 |  |  | 12.3 | 9.89 |
|  | p | 0.486 | 0.00128 | 0.00017 | 2.05x10^-7^ |  |  | 0.00082 | 0.00252 |
| Ni | Effect size | 0.63 ±0.075 | 0.13 ±0.10 |  | -0.27 ±0.092 | -0.07 ±0.031 |  | -0.20 ±0.12 |  |
|  | F(1, 66) | 9.2 | 10.8 |  | 33.5 | 16.8 |  | 10.7 |  |
|  | p | 0.00345 | 0.00167 |  | 2.18x10^-7^ | 0.00011 |  | 0.00171 |  |
| Pb | Effect size | 0.89 ±0.039 |  | -0.1 ±0.039 | 0.04 ±0.043 |  |  |  |  |
|  | F(1, 68) | 183 |  | 25.6 | 3.75 |  |  |  |  |
|  | p | 0 |  | 3.41x10^-6^ | 0.057 |  |  |  |  |

P_settling_ = post-treatment, settling only; E_Fe_ = electrode material, iron; H_6.5_ = pH 6.5; G = conductivity (mS/cm)

**Table B.2**: Regression models for residual coagulant metal concentrations, in mg/L. Plus and minus values for effect sizes represent 95% confidence intervals.

| Metal |  | Intercept | P_settling_ | H_6.5_ | G  (mS/cm) |
| --- | --- | --- | --- | --- | --- |
| Al | Effect size (mg/L) | 2.6 ±1.4 | 2.1 ±1.4 | -2.4 ±1.5 |  |
|  | F(1, 32) | 13.4 | 8.80 | 9.82 |  |
|  | p | 0.000883 | 0.00566 | 0.00368 |  |
| Fe | Effect size (mg/L) | -1.5 ±1.3 | 4.1 ±1.5 |  | 2.0 ±0.78 |
|  | F(1, 33) | 5.11 | 28.2 |  | 23.8 |
|  | p | 0.0305 | 7.39x10^-6^ |  | 2.64x10^-5^ |

P_settling_ = post-treatment, settling only; H_6.5_ = pH 6.5; G = conductivity (mS/cm)

**References**

1. NSF/ANSI. *NSF/ANSI 53 - 2011a Drinking Water Treatment Units - Health Effects*. (2011).

2. Snoeyink, V. L. & Jenkins, D. *Water Chemistry*. (John Wiley & Sons, Inc., 1980).
